# Reconstructive types effect the prognosis of patients with tumors in the central and nipple portion of breast cancer? An analysis based on SEER database

**DOI:** 10.3389/fonc.2022.1092506

**Published:** 2023-01-23

**Authors:** Ping Wang, Le Wang, Xiaming Liang, Erran Si, Yongguang Yang, Lingfei Kong, Yonghui Dong

**Affiliations:** ^1^ Department of Pathology, Henan Provincial People’s Hospital, Zhengzhou University People’s Hospital, Henan University People’s Hospital, Zhengzhou, China; ^2^ Department of Nephrolgy, Tongji Hospital, Tongji Medical College, Huazhong University of Science and Technology, Wuhan, Hubei, China; ^3^ Department of Orthopedics, Henan Provincial People’s Hospital, Zhengzhou University People’s Hospital, Henan University People’s Hospital, Zhengzhou, China; ^4^ Central Catheter Room, Henan Provincial People’s Hospital, Zhengzhou University People’s Hospital, Henan University People’s Hospital, Zhengzhou, China; ^5^ Department of Research Management, Henan Provincial People’s Hospital, Zhengzhou University People’s Hospital, Henan University People’s Hospital, Zhengzhou, China

**Keywords:** reconstructive types, prognosis, breast cancer, SEER, implant

## Abstract

**Introduction:**

The impact of different types of reconstruction, including tissue reconstruction, implant reconstruction and combined reconstruction, on patient survival were not illustrated completely. We tried to investigate the impact of patient survival between different types of reconstruction.

**Methods:**

We enrolled 6271 patients with tumors in the central and nipple portion of breast cancer from the Surveillance, Epidemiology, and End Results database. Factors associated with survival were identified by Cox regression analyses. The mortality rates per 1,000 person-years were calculated and compared. Survival curves were produced by Kaplan-Meier analyses using log-rank tests and cox proportional hazards regression quantified the risk of survival.

**Results:**

Reconstructive types, region, insurance, race, marial status, grade, stage, ER status, PR status, HER-2 status and chemotherapy were significant prognostic factors associated with breast cancer-specific survival. The breast cancer mortality rates per 1,000 person-years for patients with tissue, implant and combined group were 26.01,21.54 and 19.83 which showed a downward trend. The HR of implant and combined reconstruction adjusted for demographic, pathological, and therapeutic data was 0.82 (95% CI: 0.67-1.00, p=0.052) and 0.73(95% CI:0.55-0.97, p=0.03) compared with tissue reconstruction.

**Conclusion:**

Breast cancer-related mortality between implant reconstruction and autologous tissue reconstruction showed no significantly different, but the risk of BCSS of compound reconstruction was lower than tissue reconstruction.

## Introduction

Breast cancer is the most common cause of female malignant tumor-related death worldwide, and its morbidity and mortality are on the rise (1, 2). In addition to survival after surgery, chemotherapy and radiotherapy (3), aesthetic outcomes and quality of life after breast cancer treatment is also a consideration by breast surgeons and plastic surgeons today (4–7).

Reconstruction mitigated the psychosocial and physical consequences of mastectomy by restoring the breast mound. Of the 252,710 women estimated to have been diagnosed with invasive breast cancer in the United States in 2017, more than a third of patients with early-stage disease would opt for mastectomy as their primary surgical procedure. For these women, plastic surgeons may opt for reconstruction (8, 9).

The types of breast reconstruction mainly include implant breast reconstruction, autologous breast reconstruction and compound reconstruction. The current data focus on the potential risk of complications and long-term satisfaction among patients with different types of breast reconstruction, and help future patients understand the existing breast reconstruction options. This decision is based on expected satisfaction with the breast and quality of life. However, there is rare studies on the impact of different types of reconstruction, including compound reconstruction, on patient survival. Here, we try to conduct on this issue using a large sample of data.

## Methods

### Data source and cohort ascertainment

The Surveillance, Epidemiology and End Results (SEER) database contains demographic, pathological and treatment characteristics on cancer patients which collected and provided by the National Cancer Institute (https://seer.cancer.gov/), and is used as the source of data for our study. We recruited breast cancer patients from SEER database by using code C50.0 (Nipple) and C50.1 (Central portion of breast) from the International Classification of Diseases for Oncology.

The following demographic information and clinical characteristics were collected: age at diagnosis (2010-2016), region, insurance, year of diagnosis, sex, race, marial status, laterality, extension, grade, AJCC Stage, ER status, PR status, HER2 status, radiotherapy, chemotherapy, and reconstructive method. To ensure the accuracy of the results, user missing value was performed for missing or unknown data.

### Statistical analysis

Quantitative variables are expressed as median (interquartile range), while categorical variables are presented as percentages. We used univariate Cox regression analysis to investigate clinicopathological factors associated with breast cancer-specific mortality, and compared breast cancer-specific and all-cause mortality rates per 1000 person-years in patients with different types of reconstruction. Kaplan-Meier analysis was performed using log-rank test to evaluate the difference in survival risk among different risk factors. Finally, the effects of different groups on specific and all-cause mortality were assessed using a multivariate Cox regression analysis, adjusted for demographic, pathological, and therapeutic characteristics. All p-values were two-sided, with p < 0.05 considered statistically significant. Data extraction was processed by official software “SEERStat” version 8.3.7. Statistical analyses were performed using SPSS version 24.0 (IBM Corp., Armonk, NY), GraphPad Prism version 7 (GraphPad Software Inc., La Jolla, CA) and Stata/SE version 15 (Stata Corp., College Station, TX).

## Results

### General characteristics of the study population

Of the 6271 patients with tumors in the central and nipple portion of breast cancer, tissue reconstructive group with insurance occupied 2116(34.69%), implant and combined reconstructive group 3005(49.27) and 978(16.04), (p=0.13) respectively. Median age were 49.00, 47.00 and 49.00 (p=0.02) in tissue, implant and combined reconstructive group, respectively. According to the TNM-8^th^ system, 143 patients (35.05%) with stage I, 1048 (34.04%) with stage II, 953 (35.89%) in stage III and 48 (37.21%) in stage IV were placed in group with tissue reconstruction, respectively; 197 patients (48.28%) with stage I, 1540 (50.02%) with stage II, 1281 (48.25%) in stage III and 59 (45.74%) in stage IV were placed in group with implant reconstruction, respectively; in addition, 68 patients (16.67%) with stage I, 491(15.94%) with stage II, 421(15.86%) in stage III and 22 (17.05%) in stage IV were placed in group with implant reconstruction, respectively. Other demographic, clinicopathological and therapy characteristics are presented in **Table 1**.

**Table 1:** Clinicopathological features of the central and nipple breast cancer patients.

Variables	Tissue N (%)	Implant N (%)	Combined N (%)	P value
Region				<0.01
Alaska	4(57.14)	2(28.57)	1(14.29)	
East	1175(39.22)	1367(45.63)	454(15.15)	
Northen	204(23.23)	423(48.18)	251(28.59)	
Pacific	691(33.70)	1102(53.76)	257(12.54)	
Southwest	118(34.71)	183(53.82)	39(11.47)	
Insurance				0.13
NO	46(46.00)	41(41.00)	13(13.00)	
Yes	2116(34.69)	3005(49.27)	978(16.04)	
Age	48.78( ± 10.52)	48.37( ± 11.18)	49.15( ± 10.55)	0.04
Median (interquartile range)	49.00(41.00-56.00)	47.00(40.00-56.00)	49.00(41.75-57.00)	0.02
Race				<0.01
White	1680(33.76)	2486(49.96)	810(16.28)	
Black	344(42.16)	347(42.52)	125(15.32)	
Other	155(33.99)	237(51.97)	64(14.04)	
Marial status				0.37
Married	1414(34.24)	2043(49.47)	673(14.97)	
Single	371(37.06)	480(47.96)	150(14.97)	
Divorced/Separated/Widowed	314(34.47)	451(49.51)	146(16.02)	
Year of diagnosis				0.89
2010-2013	1374(34.91)	1939(49.26)	623(15.83)	
2014-2016	818(35.03)	1138(48.74)	379(16.23)	
Laterality				0.22
left	1092(35.41)	1503(48.74)	489(15.85)	
right	1100(34.53)	1574(49.40)	512(16.07)	
Grade				0.85
I	187(31.22)	312(52.09)	100(16.69)	
II	934(34.67)	1340(49.74)	420(15.59)	
III	987(35.88)	1318(47.91)	446(16.21)	
IV	5(35.71)	6(42.86)	3(21.43)	
AJCC^a^ Stage				0.80
I	143(35.05)	197(48.28)	68(16.67)	
II	1048(34.04)	1540(50.02)	491(15.94)	
III	953(35.89)	1281(48.25)	421(15.86)	
IV	48(37.21)	59(45.74)	22(17.05)	
ER status				0.49
Negative	459(36.34)	610(48.30)	194(15.36)	
Positive	1733(34.60)	2467(49.26)	808(16.13)	
PR status				0.35
Negative	708(36.18)	936(47.83)	313(15.99)	
Positive	1484(34.40)	2141(49.63)	689(15.97)	
HER2 status				0.23
Negative	1669(34.40)	2403(49.52)	780(16.08)	
Positive	523(36.86)	674(47.50)	222(15.64)	
Radiotherapy				0.36
YES	75(30.61)	135(55.10)	35(14.29)	
NO	2115(35.14)	2937(48.80)	966(16.05)	
Chemotherapy				0.89
YES	239(34.49)	346(49.93)	108(15.58)	
NO	1953(35.01)	2731(48.96)	894(16.03)	

a AJCC, American Joint Committee on Cancer.

### Clinicopathological factors associated with breast cancer specific survival and overall survival

In the univariate Cox regression analysis, reconstructive types, region, insurance, race, marial status, grade, stage, ER status, PR status, HER-2 status and chemotherapy were significant prognostic factors of BCSS (all, p < 0.05), age, year of diagnosis, laterality and radiotherapy did not show significant difference of BCSS (**Table 2**). Univariate Cox analyses of the factors associated with OS showed similar results (**Table 2**).

**Table 2:** Univariable Cox proportional hazard regression model of breast cancer-specific survival (BCSS) and overall survival (OS).

Variables	BCSS		OS	
	HR (95% CI)	P†	HR (95% CI)	P†
Reconstruction				
Tissue	Ref		Ref	
Impant	0.80(0.66-0.97)	0.03	0.82(0.68-0.99)	0.04
Combined	0.73(0.55-0.97)	0.03	0.75(0.57-0.99)	0.04
Region				
Alaska	Ref		Ref	
East	0.31(0.08-1.24)	0.10	0.34(0.08-1.36)	0.13
Northen	0.25(0.06-1.00)	0.05	0.27(0.07-1.10)	0.07
Pacific	0.25(0.06-1.01)	0.05	0.26(0.07-1.06)	0.06
Southwest	0.29(0.07-1.22)	0.09	0.30(0.07-1.27)	0.10
Insurance				
NO	Ref		Ref	
Yes	0.50(0.29-0.87)	0.02	0.54(0.31-0.94)	0.03
Age	1.00(0.99-1.01)	0.67	1.01(1.00-1.02)	0.08
Race				
White	Ref		Ref	
Black	1.78(1.42-2.24)	<0.01	1.80(1.45-2.24)	<0.001
Other	0.77(0.51-1.17)	0.77	0.75(0.50-1.12)	0.16
Marial status				
Married	Ref		Ref	
Single	1.58(1.25-2.00)	<0.01	1.57(1.25-1.96)	<0.001
Divorced/Separated/Widowed	1.32(1.03-1.70)	0.03	1.33(1.04-1.69)	0.02
Year of diagnosis				
2010-2013	Ref		Ref	
2014-2016	0.92(0.69-1.23)	0.56	0.92(0.69-1.22)	0.55
Laterality				
left	Ref		Ref	
right	1.02(0.85-1.23)	0.82	1.04(0.87-1.23)	0.70
Grade				
I	Ref		Ref	
II	2.02(1.16-3.51)	0.01	1.65(1.02-2.67)	0.04
III	5.07(2.97-8.67)	<0.01	3.95(2.49-6.28)	<0.001
IV	8.49(2.44-29.53)	<0.01	6.23(1.84-21.06)	<0.01
AJCC^a^ Stage				
I	Ref		Ref	
II	2.02(1.16-3.51)	0.01	1.38(0.84-2.28)	0.21
III	5.07(2.97-8.67)	<0.01	2.95(1.81-4.82)	<0.01
IV	8.49(2.44-29.53)	<0.01	12.21(6.94-21.46	<0.01
ER status				
Negative	Ref		Ref	
Positive	0.32(0.27-0.38)	<0.01	0.34 (0.28-0.40)	<0.001
PR status				
Negative	Ref		Ref	
Positive	0.29(0.24-0.34)	<0.01	0.31(0.26-0.37)	<0.001
HER2 status				
Negative	Ref		Ref	
Positive	0.70(0.59-0.89)	<0.01	0.71(0.56-0.89)	<0.01
Radiotherapy				
No/Refused	Ref		Ref	
Beam	1.27(0.73-2.20)	0.40	0.98(0.61-1.57)	0.92
Radioisotopes	1.67(0.22-12.80)	0.62	1.20(0.16-9.00)	0.86
Chemotherapy				
YES	Ref		Ref	
NO	2.07(1.39-3.07)	<0.01	1.86(1.29-2.67)	<0.01

a AJCC, American Joint Committee on Cancer.

^†^P < 0.05 was considered statistically significant.

### Breast cancer mortality and overall mortality rates per 1,000 person-years

During the follow-up till December 2016, the BCM rates per 1,000 person-years for patients with tissue, implant and combined group were 24.55,19.80 and 18.15. Moreover, the breast cancer mortality rates per 1,000 person-years for patients with tissue, implant and combined group were 26.01,21.54 and 19.83, all groups showed a downward trend (**Table 3**).

**Table 3:** Comparison of breast cancer and overall mortality per 1,000-person-year between different reconstruction types.

	Breast cancer mortality per 1,000-person-year			overall mortality per 1,000-person-year		
	Fail	Rate	95% CI	Fail	Rate	95% CI
Recontruction						
Tissue	184	24.55	21.24-28.36	195	26.01	22.61-29.93
Implant	215	19.80	17.32-22.63	234	21.54	18.95-24.49
Combined	65	18.15	14.23-23.15	71	19.83	15.71-25.02

CI, confidence interval.

### Hazard ratios of different subgroups for BCSS

The HRs for BCSS of the group with tissue reconstruction compared with the other groups are displayed in **Table 4**. The unadjusted HR of the group with implant reconstruction was 0.83 (95% CI: 0.68-1.01, p=0.56) compared with group with tissue reconstruction; The HR adjusted for demographic data was 0.83(95% CI: 0.68-1.00, p=0.06). The HR adjusted for demographic and pathological data was 0.84(95% CI: 0.69-1.03, p=0.10). The HR adjusted for demographic, pathological, and therapeutic data was 0.82 (95% CI: 0.67-1.00, p=0.052). The unadjusted HR of the group with combined reconstruction was 0.74(95% CI: 0.56-0.99, p=0.04) compared with group with tissue reconstruction; As to the group with combined reconstruction, the Cox regression HRs for adjusted 1, adjusted 2, and adjusted 3 models were 0.75(95% CI:0.57-0.99), p=0.05, 0.73(95% CI:0.55-0.97), p=0.03, and 0.75(95% CI:0.56-0.99), p=0.04, respectively.

**Table 4:** Hazard ratios of different reconstruction types for the cancer specific mortality of breast cancer.

	Unadjusted Cox regression		Adjusted 1 Cox regression		Adjusted 2 Cox regression		Adjusted 3 Cox regression	
	Hazard Ratio (95% CI)	p- value	Hazard Ratio (95% CI)	p- value	Hazard Ratio (95% CI)	p- value	Hazard Ratio (95% CI)	p- value
Reconstruction								
Tissue	ref		Ref		Ref		Ref	
Implant	0.83(0.68-1.01)	0.56	0.83(0.68-1.00)	0.06	0.84(0.69-1.03)	0.10	0.82(0.67-1.00)	0.052
combined	0.74(0.56-0.99)	0.04	0.75(0.57-0.99)	0.05	0.73(0.55-0.97)	0.03	0.75(0.56-0.99)	0.04

Adjusted 1 Cox regression: cox regression for year at diagnosis and race matched reconstruction types.

Adjusted 2 Cox regression: cox regression for year at diagnosis, race stage, ER status, PR status and HER2 status matched reconstruction types.

Adjusted 3 Cox regression: cox regression for age at diagnosis, year at diagnosis, sex, race, stage, ER status, PR status, HER2 status, radiation therapy and chemotherapy matched reconstruction types.

### Hazard ratios of different subgroups for OM

The HRs for OM of the group with tissue reconstruction compared with the other groups are displayed in **Table 5**. The unadjusted HR of the group with implant and combined reconstruction were 0.82 (95% CI: 0.68-0.99, p=0.04) and 0.75(95% CI: 0.57-0.99, p=0.04) compared with group with tissue reconstruction, respectively. The HR adjusted for demographic data with implant and combined reconstruction were 0.85(95% CI: 0.70-1.03, p=0.09) and 0.77(95% CI: 0.58-1.01, p=0.054). The HR adjusted for demographic and pathological data with implant and combined reconstruction were 0.85(95% CI: 0.70-1.02, p=0.09) and 0.78(95% CI:0.59-1.02, p=0.07). The HR adjusted for demographic, pathological, and therapeutic data with implant and combined reconstruction were 0.84 (95% CI: 0.70-1.02, p=0.08) and 0.77(95% CI: 0.59-1.01, p=0.06), respectively.

**Table 5:** Hazard ratios of different reconstruction types for all-cause mortality of breast cancer.

	Unadjusted Cox regression		Adjusted 1 Cox regression		Adjusted 2 Cox regression		Adjusted 3 Cox regression	
	Hazard Ratio (95% CI)	p- value	Hazard Ratio (95% CI)	p- value	Hazard Ratio (95% CI)	p- value	Hazard Ratio (95% CI)	p- value
Reconstruction								
Tissue	ref		Ref		Ref		Ref	
Implant	0.82(0.68-0.99)	0.04	0.85(0.70-1.03)	0.09	0.85(0.70-1.02)	0.09	0.84(0.70-1.02)	0.08
combined	0.75(0.57-0.99)	0.04	0.77(0.58-1.01)	0.054	0.78(0.59-1.02)	0.07	0.77(0.59-1.01)	0.06

Adjusted 1 Cox regression: cox regression for year at diagnosis and race matched reconstruction types.

Adjusted 2 Cox regression: cox regression for year at diagnosis, race stage, ER status, PR status and HER2 status matched reconstruction types.

Adjusted 3 Cox regression: cox regression for age at diagnosis, year at diagnosis, sex, race, stage, ER status, PR status, HER2 status, radiation therapy and chemotherapy matched reconstruction types.

### Kaplan-Meier analyses using log-rank tests

**Figure 1:**
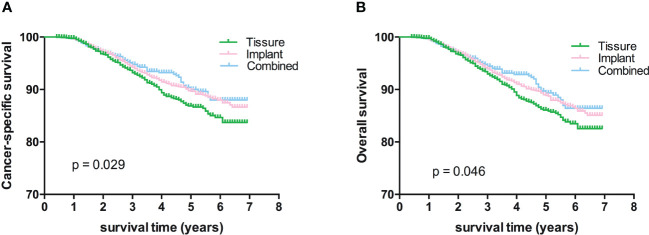
Kaplan-Meier analyses of CSS **(A)** and OS **(B)** among patients stratified according to reconstructive types.

**Figure 2:**
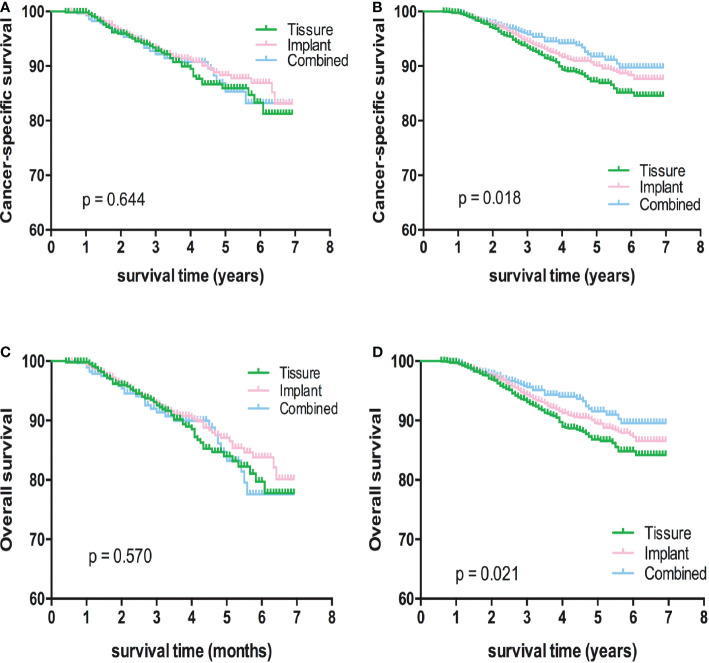
Kaplan-Meier analyses of CSS **(A, B)** and OS **(C, D)** among patients over or under 55 years stratified according to reconstructive types.

**Figure 3:**
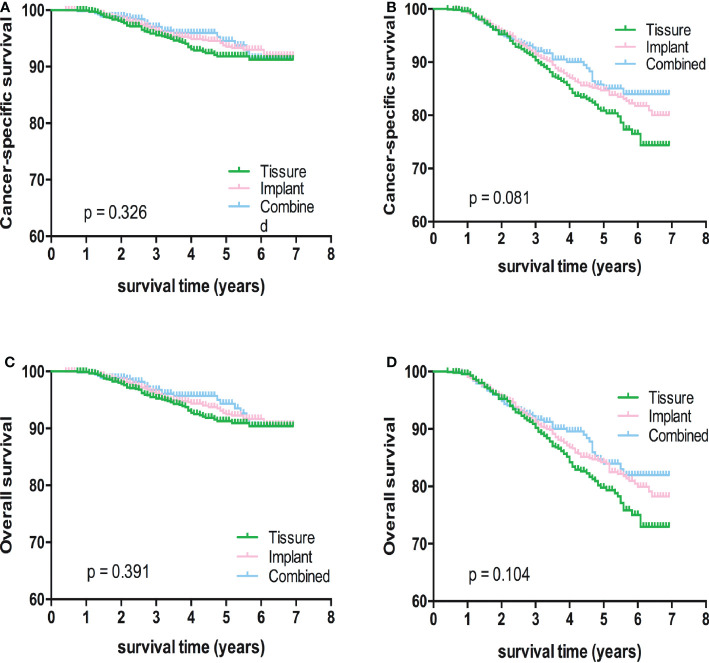
Kaplan-Meier analyses of CSS **(A, B)** and OS **(C, D)** among patients with I/II or III/IV stage stratified according to reconstructive types.

**Figure 4:**
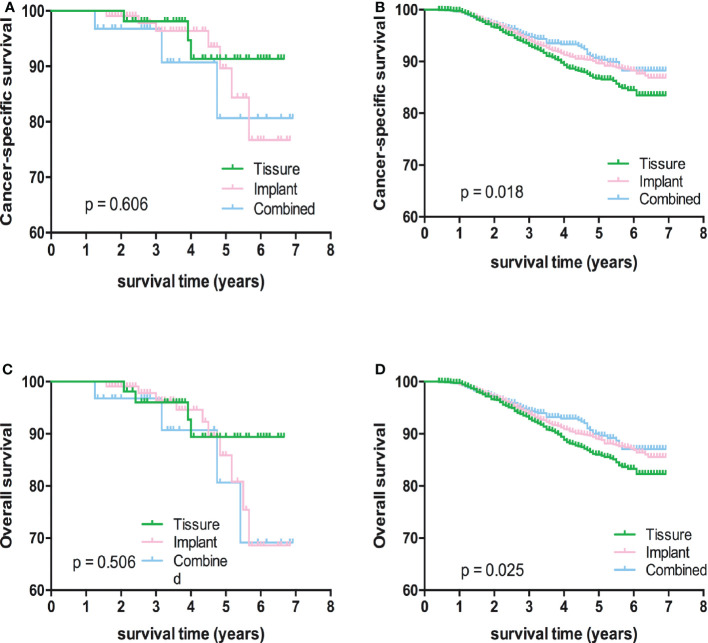
Kaplan-Meier analyses of CSS **(A, B)** and OS **(C, D)** among patients with or without radiotherapy stratified according to reconstructive types.

Kaplan-Meier analyses using log-rank tests showed that BCSS and OS were significantly different between the reconstructive types groups (**Figures 1A, B**). Meanwhile, BCSS and OS with the cases under 55 years also showed significantly different between the reconstructive types groups (**Figures 2B, D**). In addition, BCSS and OS with the cases with radiotherapy were significantly different between reconstructive types groups (**Figures 4B, D**). Prognosis of cases with different stage, over 55 years and without radiotherapy were showed no significant difference between different reconstructive types groups (**Figures 2**–**4**).

## Discussion

In this study, we tried to conduct on the impact of different types of reconstruction, including tissue reconstruction, implant reconstruction and combined reconstruction, on patient survival using SEER database, and found that group with combined reconstruction showed less Hazard Ritio:0.75(95% CI:0.56-0.99) than the tissue reconstruction group for the breast cancer specific mortality. And between tissue and implant reconstructive group, the BCSS were not significantly different.

Breast reconstruction has become common in breast cancer surgery (10–12). In the United States, breast reconstruction rates are increasing among early-stage breast cancer patients who undergo mastectomies, most of which involve implant reconstruction, as well as reconstruction of autologous tissue. And the more severe the defect, the higher the rate of reconstruction, for example, about three-quarters of patients who had bilateral mastectomies had breast reconstruction at the same time (9).

Autogenous tissue breast reconstruction is the use of a patient’s own tissue, transplanted from other parts of the body, to the breast area to restore breast volume after mastectomy. Common autologous tissue reconstruction methods include muscle flaps of rectus abdominis (TRAM), deep abdominal perforator flaps (DIEP), latissimus dorsi flap, autologous fat transplantation. Autogenous breast reconstruction is considered the gold standard by many plastic surgeons because it is softer, does not carry risks such as capsular contracture, and has a better aesthetic effect (13, 14).

Although radiation therapy increases the rate of capsular contracture and reconstruction failure of implants, implant breast reconstruction is a practical option, especially for some patients who lack autologous tissue (4, 15, 16). Regardless of the type of reconstruction, ensuring the safety of the patient’s tumor reconstruction is a top priority for clinicians and patients. In our study, there was no difference in cancer-related mortality after adjusting for all potential influencing factors between implant reconstruction and autologous tissue reconstruction, but the risk of compound reconstruction was lower. Pusic et al. reported that the satisfaction with breasts, psychosocial well-being, and sexual well-being of autologous breast reconstruction were significantly higher than that of implant reconstruction (17–19), Therefore, in this perspective, autologous breast reconstruction can be more cost-effective without affecting survival outcomes.

In patients with advanced breast cancer, postoperative radiotherapy has been shown to improve patient survival, especially in patients with positive lymph nodes (20). But it has some negative consequences for women with breast reconstruction, such as complications and reduced cosmetic results. With advances in plastic surgery and radiation therapy, clinicians are already trying to integrate radiation therapies to minimize interference with the effects of reconstruction with minimal impact on tumor survival (9). In our study based on SEER big data, we found that the rate of implant reconstruction is higher than tissue reconstruction for the patients underwent radiotherapy.

As with all large database studies, our study still has some limitations. First, genetic factors such as BRCA mutations should be considered in comparative analysis. Secondly, our data did not contain specific information about immediate reconstruction or delayed reconstruction, nipple-sparing mastectomy or not, and there was no detailed record of complications of reconstructive surgery. Finally, the population we included is based on SEER database, which mainly includes the population of North America. These may affect the generality of our systematic conclusions.

## Conclusion

Breast cancer-related mortality between implant reconstruction and autologous tissue reconstruction showed no significantly different, but the risk of BCSS of compound reconstruction was lower than tissue reconstruction.

## Data availability statement

The original contributions presented in the study are included in the article/**Supplementary Material**. Further inquiries can be directed to the corresponding authors.

## Author contributions

PW wrote the main manuscript text. YD and LW edited and revised the article critically. XL did the literature research, and ES collected the data. LK provided the source of data information, YY analyzed the data, and PW prepared figures. YD and PW provided the study concepts and study design. All authors contributed to the article and approved the submitted version.
